# Optoregulated force application to cellular receptors using molecular motors

**DOI:** 10.1038/s41467-021-23815-4

**Published:** 2021-06-11

**Authors:** Yijun Zheng, Mitchell K. L. Han, Renping Zhao, Johanna Blass, Jingnan Zhang, Dennis W. Zhou, Jean-Rémy Colard-Itté, Damien Dattler, Arzu Çolak, Markus Hoth, Andrés J. García, Bin Qu, Roland Bennewitz, Nicolas Giuseppone, Aránzazu del Campo

**Affiliations:** 1 INM – Leibniz Institute for New Materials, Saarbrücken, Germany; 2 Biophysics, CIPMM, School of Medicine, Saarland University, Homburg, Germany; 3 Woodruff School of Mechanical Engineering, Georgia Institute of Technology, Atlanta, GA USA; 4 Petit Institute for Bioengineering and Bioscience, Georgia Institute of Technology, Atlanta, GA USA; 5 SAMS Research Group, Institut Charles Sadron, University of Strasbourg – CNRS, Strasbourg, France; 6 Saarland University, Physics Department, Saarbrücken, Germany; 7 Saarland University, Chemistry Department, Saarbrücken, Germany

**Keywords:** Nanoscale biophysics, Molecular machines and motors

## Abstract

Progress in our understanding of mechanotransduction events requires noninvasive methods for the manipulation of forces at molecular scale in physiological environments. Inspired by cellular mechanisms for force application (i.e. motor proteins pulling on cytoskeletal fibers), we present a unique molecular machine that can apply forces at cell-matrix and cell-cell junctions using light as an energy source. The key actuator is a light-driven rotatory molecular motor linked to polymer chains, which is intercalated between a membrane receptor and an engineered biointerface. The light-driven actuation of the molecular motor is converted in mechanical twisting of the entangled polymer chains, which will in turn effectively “pull” on engaged cell membrane receptors (e.g., integrins, T cell receptors) within the illuminated area. Applied forces have physiologically-relevant magnitude and occur at time scales within the relevant ranges for mechanotransduction at cell-friendly exposure conditions, as demonstrated in force-dependent focal adhesion maturation and T cell activation experiments. Our results reveal the potential of nanomotors for the manipulation of living cells at the molecular scale and demonstrate a functionality which at the moment cannot be achieved by other technologies for force application.

## Abstract (web summary)

Molecular scale force application in physiological environments is important for studying mechanotransduction. Here, the authors use a molecular machine to apply forces at cell-matrix and cell-cell junctions using light to trigger twisting actuation which then pulls on cell membrane receptors.

## Introduction

External mechanical stimuli are sensed and translated by cells into biochemical signals in a process called mechanotransduction^1,2^. In turn, biochemical signals regulate cellular and extracellular mechanical properties. This mechano-sensitive feedback modulates cellular functions as diverse as proliferation, differentiation, migration, and apoptosis, and is crucial for tissue formation, homeostasis, repair, and pathogenesis. Perturbations along the chain of mechanical sensing and biochemical responses lead to various pathological disorders such as loss of hearing, cardiovascular dysfunction, muscular dystrophy, and cancer. Being able to apply mechanical signals to cells at the molecular scale in cell culture models or tissues is of great value in the understanding of these diseases.

Cells generate forces at the molecular scale through directional polymerization of actin filaments and by the action of myosin as ATP-driven molecular motor^3^. The objective of the present study focuses on the application of external forces to cells using an artificial, purely synthetic molecular motor^4^. Among synthetic molecular devices, light-driven rotary motors based on over-crowded alkene molecules are able to autonomously cycle unidirectional 360° rotations around their central double bond by using light and temperature as combined sources of energy^5^. When rotary motors are coupled to fixed elements through a number of polymer chains, the rotational actuation of the motor forces conformational twisting and thus an increasing entanglement of the polymer chains. In this configuration, the original rotary motion is transformed into a contraction of the chain ensemble, with the potential to pull on individual nanoscale objects^4,6–9^.

In this work, we envisioned using molecular motor-polymer conjugates to apply external mechanical forces to individual membrane receptors of a living cell, and to trigger mechanotransduction processes therefrom. We demonstrate the implementation and application of this molecular tool in two relevant mechanotransduction scenarios: force-dependent focal adhesion (FA) maturation and force-dependent T-cell activation. Furthermore, we show that motor-polymer ensembles apply forces in the range of tens of pN, by tracking the light-induced pulling of microparticles tethered to motor-chain conjugates against the drag force of liquid flow in a microfluidic channel.

## Results and discussion

### Motor substrate functionalization

**Fig. 1:**
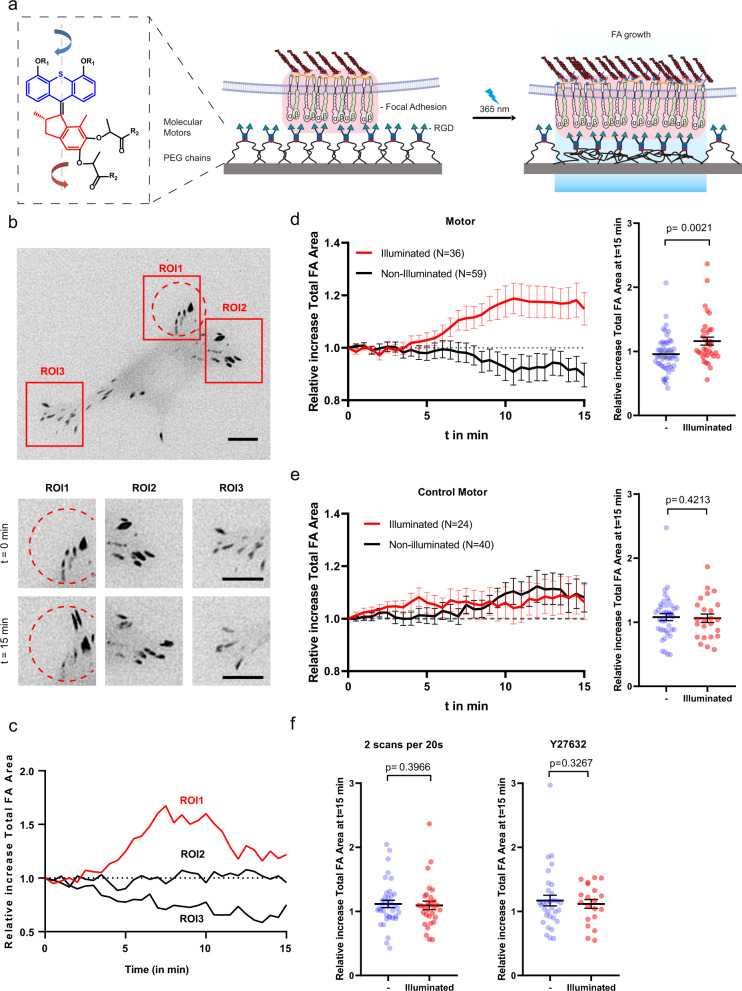
Light-driven force application by the motor substrate coupled to RGD integrin-binding ligands drives focal adhesion growth and maturation. **a** Scheme showing the design of the force application platform with the rotary motor. The red box represents the motor-RGD-Integrin cell adhesion interface within focal adhesions. **b** Representative image of focal adhesions in MEF expressing paxillin-RFP adhering to the molecular motor-RGD substrate tracked over time during substrate activation using UV-light scans. The activated area is demarcated by the red dotted lines. Scale bar = 10 µm. **c** Higher magnification images of the ROIs at time points *t* = 0 min and 15 min (left) and the effect of irradiation on the total FA area were analyzed within defined ROIs (red boxes in **b**). **d** Plots represent the mean ± s.e.m. of relative increase in total FA area of analyzed within *n* ROIs of cells seeded on motor substrate illuminated with 5 cycles per 20 s. The scatterplot on the right represents the relative increase in total FA area at *t* = 15 min with the result from each ROI plotted individually. ROIs were analyzed from 34 cells from six independent experiments. Non-illuminated ROIs (*n* = 59) and illuminated ROIs (*n* = 34). **e** Same as in (**d**) but cells were seeded on the control motor substrate. *n* ROIs from 24 cells from five independent experiments. Non-illuminated ROIs (*n* = 40), and illuminated ROIs (*n* = 24). **f** Scatterplots (mean ± s.e.m.) with each datapoint representing an analyzed ROI at *t* = 15 min. Cells were UV-illuminated with 2 scans per 20 s (left—30 cells from three independent experiments—non-illuminated ROIs (*n* = 59), and illuminated ROIs (*n* = 30)) or with 5 scans per 20 s but pre-treated with ROCK inhibitor Y27632 (right—20 cells from three independent experiments—non-illuminated ROIs (*n* = 32), and illuminated ROIs (*n* = 20)). Data from non-illuminated and illuminated areas within each condition were compared using an unpaired one-tailed Student’s *t*-test.

The general molecular design used for the present study includes a light-driven molecular motor with a so-called stator part (in blue) and a rotor part (in red) (Fig. 1a and Supplementary Fig. 1). The stator part is linked to two triethylene glycol (TEG) chains connected to the membrane receptor of interest (e.g., integrin or T-cell receptor) via a complementary ligand (i.e., RGD or anti-CD3). Although the exact binding configuration at the membrane receptor cluster is unknown, we expect that multivalency of the motor surface stabilizes the connection between integrins and the short spacer in view of a short-lived receptor-ligand bond. The rotor part is linked to two flexible polyethylene glycol (PEG) chains (*M*_w_ ≈ 5000 g.mol^−1^) and is connected to the substrate by covalent bonds. The orthogonal chemical protocols used for the two coupling steps (i.e., catalyzed and non-catalyzed click reactions) are selective and, therefore, no cross-reactions are expected (Supplementary Fig. 1). In this configuration, light irradiation progressively rotates the motor that twists the two pairs of polymer chains and effectively shortens the receptor-interface link by increasing the entanglement of connecting polymer chains. As a consequence, a tensional force is applied directly on the membrane receptor, presumably engaged with the contractile cytoskeletal machinery. The entanglement of connecting chains is facilitated by the torque of the molecular motor and, therefore, the entropic force that originates in the reduced configurational space after entanglement may also have a tangential component. Previous studies have demonstrated that the energy produced by a single motor in a similar polymer conjugate is in the range of 12 kT (or 50 pN·nm), with a typical frequency of rotation in the range of 1–0.01 Hz at room temperature^6,7^. This range of forces and time scales seems appropriate for applying forces to cells.

In order to demonstrate the specific and functional coupling of the RGD ligand to the motor/PEG/surface, cell adhesion experiments were performed. L929 fibroblasts were seeded on the motor/PEG/surface and on the RGD/motor/PEG/surface conjugates. No cell attachment was observed on the motor/PEG/surface (Supplementary Fig. 2a) or on the substrates incubated with RGD in absence of the motor (Supplementary Fig. 2b). Cells attached and spread on RGD/motor/PEG/surfaces and showed normal morphology on the substrates over 7 days (Supplementary Fig. 2c), indicating that the spacer-motor conjugate was not toxic to the cells. Cells did not attach to substrates modified with the negative control peptide, RDG/motor/PEG/surface (Supplementary Fig. 2d). The density of attached and spread cells increased with the RGD density on the surface, which was regulated by the incubation concentration of RGD ligand in the surface modification step (Supplementary Fig. 3). These results demonstrate that (i) the coupling reaction between motors and RGD is specific, (ii) only RGD mediates cell binding to the substrates, and (iii) binding of RGD ligand to the surface is mediated solely by the motor conjugate.

### Motor-induced focal adhesion growth

After optimization of the coupling reactions for specific and stable cell binding to the surface-immobilized motor substrates), we first sought to regulate FA growth through force application at the RGD/integrin complex. Mouse embryonic fibroblasts (MEFs) expressing paxillin-RFP to mark FAs in live cells were seeded on the RGD-coupled motor substrates and allowed to adhere overnight. We illuminated circular areas (62 μm^2^) of the cells containing several FAs using a 365 nm point-by-point scanning laser. Exposure conditions were 5 scans every 20 s for 15 min. Higher substrate irradiation dosages led to retraction of the cell arm at the location of irradiation (Supplementary Fig. 4). We tracked the total FA area in the exposed and in control (not exposed) regions of the same cell by imaging RFP-paxillin every 30 s for 15 min, a timescale which has been reported for FA growth after force application^10,11^. Figure 1b shows an example of a MEF after illumination. The FA area in the illuminated region of the cell (ROI1, Fig. 1c) increased over time with light exposure, whereas the FA area in the control, non-illuminated regions (ROI2 and ROI3, Fig. 1c) did not. Quantification of ROIs from multiple cells showed, on average, a rise in total FA area in illuminated regions starting at 5 min and an increase in total FA area of about 20% at 10 min, which is sustained until the end of the measurement. In contrast, the total FA area in non-illuminated regions from the same cells decreased to about 95% within the same timespan (Fig. 1d), showing that illumination induced a significant difference in FA growth compared to FA growth in non-illuminated areas. To rule out that UV irradiation on its own could induce the observed FA area changes, similar experiments were performed in MEF cells on control surfaces conjugated with non-rotary motors (locked by a single episulfide moiety in place of the rotating double bond, Supplementary Scheme 1). In this case, no difference in total FA area between UV-illuminated and non-illuminated regions was observed (Fig. 1e). A lower light exposure dose (2 scans per 20 s, i.e., 40% of the initial dose) did not result in measurable differences in FA area between illuminated and non-illuminated areas (Fig. 1f and Supplementary Fig. 5a), indicating that this dose (and consequently force) was not enough to trigger a cellular response. Intermittent exposure programs (i.e., 3-min on, 3-min off, in 5 scans per 20 s) did not elicit any cellular response either (Supplementary Fig. 5b, c). In addition, by perturbing actomyosin contractility via ROCK inhibition with Y27632 (10 µM), the observed motor-induced increase of the FA area was lost (Fig. 1f right, Supplementary Fig. 6), confirming the involvement of actomyosin contractility in force-induced FA reinforcement and maturation^2^. Together, these results indicate that the RGD/motor/PEG surface was able to locally apply forces on integrin-RGD complexes within FAs upon UV-light exposure, leading to downstream integrin mechanotransduction and FA area increase in an illumination dose-dependent manner. Note that the observed responses and time scales are in agreement with experimental results from established methods in mechanotransduction research.

### Motor-induced T-cell activation

**Fig. 2:**
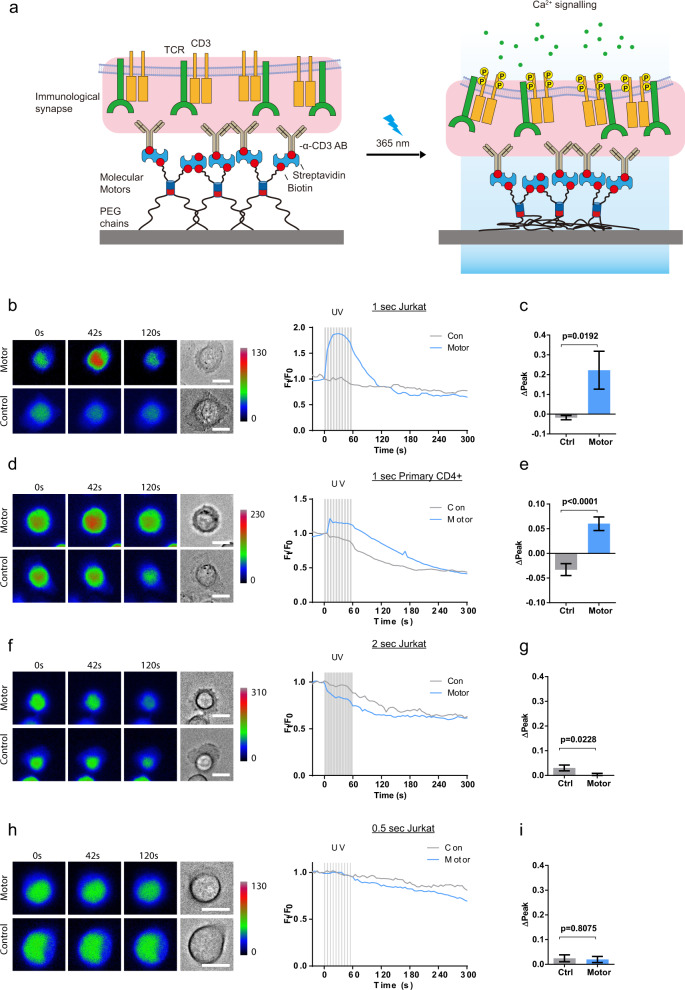
Mechanical force generated by rotatory motor can trigger TCR signaling. **a** Schematic of manufacture and activation of anti-CD3 antibody linked to the substrate via rotatable motor (Motor) or non-rotatable motor (Ctrl). The red box represents the motor-receptor interface at the immunological synapse. Ten UV pulses were applied within 60 s to activate the motor. **b**–**e** Ca^2+^ influx is induced by activation of the motor. Either Jurkat T cells (**b**, **c**) or primary human CD4^+^ T cells (**d**, **e**) loaded with Fluo-4-AM were settled on motor substrate for 20 min prior to UV illumination (starting at time 0). Duration of UV pulses was 1 s. Exemplary cells and Ca^2+^ traces are shown in (**b**) and (**d**). Ca^2+^ influx (ΔPeak) was analyzed in **c** (Ctrl, *n* = 137 cells from six independent experiments; Motor, *n* = 153 cells from nine independent experiments) and **e** (Ctrl, *n* = 42 cells from two independent experiments; Motor, *n* = 46 cells from three independent experiments). **f**–**i** Shorter or longer duration of UV pulses cannot induce Ca^2+^ influx. We used Jurkat cells and applied UV pulses with either shorter duration of 0.5 s (**f**, **g** Ctrl, *n* = 119 cells from five independent experiments; Motor, *n* = 201 cells from nine independent experiments) or extended duration of 2 s (**h**, **i** Ctrl, *n* = 101 cells from five independent experiments; Motor, *n* = 172 cells from nine independent experiments). LUT min and max given in a.u. to visually compare motor vs control substrate within the same condition only. Data in (**c**), (**g**), (**e**), and (**i**), represent mean ± s.e.m. Data from motor and control motor substrates were compared using an unpaired two-tailed Student’s *t*-test. Scale bars are 10 µm.

Mechanical stimulation of the T-cell receptor (TCR) leads to T-cell activation, as proven in single-molecule experiments^11–14^. We tested if the opto-mechanical actuator coupled to the T-cell receptor (TCR) was able to activate T cells by light exposure. For this purpose, Jurkat and primary T cells were loaded with Ca^2+^ indicator Fluo-4-AM and seeded on anti-CD3/motor/PEG-modified surface (Fig. 2a). CD3 is a key component of TCR for signal transduction, and thus Ca^2+^ influx was induced in the cells when they contacted the surface, indicating recognition of the anti-CD3 antibody. In order to decouple the Ca^2+^ influx induced by the motor from a possible response by simple interaction with the surface, Jurkat cells were seeded at 0 mM [Ca^2+^] concentration, and the same volume containing 2 mM [Ca^2+^] was added at 8 min post-seeding. At 15 min cells were exposed to pulses (1 s) of 365 nm light for 1 min (Supplementary Fig. 7). A Ca^2+^ rise was observed immediately after the first UV pulse (Fig. 2b, c), which decayed within 5 min. In comparison, no response was observed when the Jurkat cells were seeded on control motor (no rotation) surfaces exposed to UV light (Fig. 2b, c). These results indicate that the observed response was not associated to UV illumination per se, but it was a consequence of the applied pulling force on the TCR by the rotary motor linked to anti-CD3. Under the same conditions, primary human CD4^+^ T cells showed a similar response (Fig. 2d, e). Shorter light pulses (0.5 s) did not elicit an observable response (Fig. 2f, g), indicating that a certain threshold of force is required for force-mediated activation of T cells. Longer pulses (2 s) induced a decrease in the Ca^2+^ signal on the motor and control surfaces (Figs. 2h, 2i), which is most likely related to photodamage of the cells following long UV pulses. These results define the boundaries for experimentation with this unique tool. Importantly, as negative biologically relevant control, similar experiments were performed with the CD28 receptor, whose activity has been proven to be unaffected by mechanical stimulation^15^. No response was observed (Supplementary Fig. 8), confirming that the observed force-dependent Ca^2+^ response was specific to CD3 engagement. Mechanical force applied by the motor does not change the contact area at the immunological synapse (IS) (Supplementary Fig. 9). Together, these results demonstrate that synthetic molecular machines can be used to apply external forces to specific membrane receptors on T cells and study mechanotransduction events at biointerfaces. It should be noted that a similar motor had been recently incorporated into the membrane of living cells, and used to kill cells by drilling pores in the membrane upon 360-nm illumination^16^.

### Force measurements on molecular motors

**Fig. 3:**
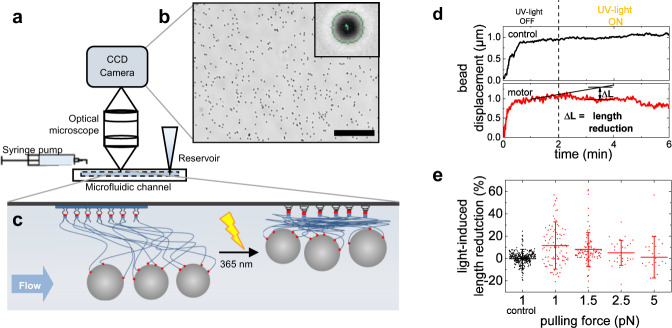
Measurement of forces applied by the light-driven rotary motor. **a** Experimental setup for the observation of tethered particle motion in a microfluidic flow channel. **b** Optical microscopy determines the movement of hundreds of surface-tethered beads in parallel (scale bar: 100 µm); the inset demonstrates tracking of one exemplary 500-nm bead. **c** Sketch of the molecular arrangement under flow before and after UV-light irradiation. Micro-beads are attached to the motor molecules via multiple DNA chains. Upon light activation, the molecular motor twists the entangled DNA chains and the construct pulls the beads in. **d** Typical bead displacement versus time after start of flow with a drag force of 1 pN. The bead displacement is suddenly reduced after the molecular motor is activated by UV light. No such reduction is observed in control experiments with the static motor molecule. **e** Average light-induced length reduction of *n* beads (mean ± standard deviation) after 2 min of UV-light irradiation for rotary motor molecule against different flow-induced drag forces and for the control with static molecules for 1 pN, normalized to the expected length of the construct (*n* = 307 (control), *n* = 97 (1 pN), *n* = 106 (1.5 pN), *n* = 28 (2.5 pN), and *n* = 26 (5 pN) obtained in five independent experiments).

In order to quantify forces and pulling distances provided by our molecular motor construct, we tracked the light-induced pulling of microparticles tethered to motor-chain conjugates against the drag force of liquid flow in a microfluidic channel (Fig. 3a–c). This tethered particle motion (TPM) method was inspired by experiments using centrifugal forces^17^. The optical quantification of the tethered microparticle motion requires spacers much longer than the PEG_5000_ linker used for the cell experiments. Therefore, we used a dsDNA chain of 1.7-µm length to replace each PEG polymer linker. The 53-fold increase in chain length is counteracted by the 36-fold increase in persistence length, rendering the twisting of entangled DNA chains a scaled version of that PEG_5000_ linkers. In the cell experiments, motor-chain conjugates were coupled to a PEG-substrate resulting in a distribution of effective tether length between receptors and surface. The heterogeneous distribution of tether lengths is expected to generate a corresponding distribution of pulling forces applied to cellular focal adhesions. In our TPM experiment, molecular motors are attached to the surface of the channel using the same substrate functionalization as in the cell experiments. The beads are connected to a small number of tethers, which resemble the attachment of focal adhesions by multiple motor-chain conjugates in the cell experiment.

Typical time-displacement curves for a microparticle are shown in Fig. 3d, details of the experiments are given in SI. In control experiments with the non-rotary motor, the bead was displaced by 1 μm in the flow direction during the first minute of flow, followed by continuous displacement (creep). UV exposure did not affect the displacement of the beads connected by the non-rotary motor. In contrast, beads tethered to the surface via entangled rotary motors were retracted against the drag force within the first minute of UV irradiation. We quantified the light-induced length reduction as ratio of the negative displacement after 2 min of illumination to the expected bead displacement at the same time point as extrapolated from creep curve before illumination. The average light-induced length reduction against four different drag forces is plotted in Fig. 3e. Against a drag force of 1 pN, the rotary motors reduced the displacement length of the tethered particles by almost 12%. This corresponds to a work per bead around 100–200 pN·nm, which is in the expected range for a few motors^6^. Length reduction decreased with increasing flow forces. These force values appear rather low compared to the tens of pN expected to be involved in mechanotransduction by integrins^18^. However, entropic forces arising from stretching of coiled chains scale inversely with their persistence length, and thus ~30-fold higher forces are expected to be applied by the twisted PEG_5000_ chains in the cell experiments, matching the relevant range for mechanical stimulation of cellular signals. We have confirmed in atomic force microscopy experiments on single molecular motors with PEG_5000_ linkers that the light-induced force of one such motor is indeed in the order of tens of pN (Supplementary Figs. 10, 11). However, single-motor experiments offer only snapshots of certain configurations and do not provide the averaging of the TPM experiment with hundreds of motors attached in the same substrate functionalization as in the cell experiment.

### Characteristic features of molecular motors vs. other force application methodologies

Much of our understanding of the molecular mechanisms underlying force-stimulated cellular processes has been garnered from studies using micropipettes^19^, atomic force microscopes (AFM)^20^, or optical tweezers^21^ to apply forces to cells at subcellular level (Table 1). The quantification of the applied forces, in parallel with the imaging of cell responses, has identified important molecules and force ranges in the mechanotransduction chain^22^. These physical methods, however, are limited in their ability to apply forces in multicellular or 3D environments, closer to physiological systems. In particular, the application of forces on integrins at the physiological cell-ECM interface is difficult, if not impossible for these techniques. Molecular approaches, like chemical-induced motors used by nature for intracellular force generation, enable a new dimension in mechanotransduction research. Salaita and co-workers ingeniously exploited a temperature-driven phase transition of a thin hydrogel film to pull on adhesive receptors of gel-bound cells^11^. This pioneering approach has, however, limitations for quantitative studies, as it does not allow gradual force regulation, and if applied in 3D cell culture, requires encapsulation of the cell in a temperature-sensitive material, restricting its application in physiologically relevant contexts. Light-driven molecular machines inserted at engineered biointerfaces, as demonstrated in the current work, allow force application directly at molecular scale, without perturbation of other interface parameters or of an external probe or a pipette. We note that the cell-substrate interface where forces are applied is normally not amenable to techniques requiring an external probe. The molecular character of the technology presented here allows positional, magnitude, and timescale regulation of the applied force by tuning light localization and intensity. Its modular design allows flexible application to any receptor/ligand complex of choice without additional synthetic effort. Table 1 compares the features of the different force application technologies.

**Table 1:** Comparison of common receptor-specific force application techniques in mechanobiology.

METHOD	Spatial resolution	Displacement range	Temporal resolution	Applied load	Loading rate	Force application interface	Special equipment	Features	Limitations
Molecular motor (this study)	±200 nm, limited by microscopy diffraction limit	1–500 nm	< second	Low pN–80 pN	0.7 pN/s (in this article)	Extracellular	365-nm light—point-by-point scanning laser for high spatial and temporal resolution	Directly incorporated at cell-substrate interface	Use of UV irradiation
								High spatiotemporal control for activation	
								Potential 3D control (not yet demonstrated)	
								Easily scalable (from subcellular to multiple cells and more)	
								Usable on most microscope setups (or even without microscope)	
								Potentially extendible to natural biointerfaces and 3D cell cultures (not yet demonstrated)	
Optical tweezers^33,34^	nm-to-μm-, depends on particle side	0.1 nm–mm	ms–min	0.1–100 pN	5–20,000 nm–mmpN/s	Intra- and extracellular	Specialized laser optics, and position detectors	High spatial control in 3D space	Heating of sample at higher force loading
								Simultaneous measurement of forces/mechanics	Low throughput (single cell)
								Localized deformations	
Magnetic tweezers^34–36^	nm-to-μm-, depends on particle size	5 nm–mm	0–1000 Hz	pN–nN	0–1000 Hz	Intra- and extracellular	(Electro)Magnetic field generator	Defined force direction (shear vs normal forces)	Finely calibrated and magnetic field control equipment needed
								Simultaneous measurement of forces/mechanics	
								Localized deformations	
								Semi high-throughput (up to multiple cells)	
AFM^37^ (single-molecule)	nm-µm	40 nm–µm	ms	pN–nN	10–1000 pN/s	Extracellular	Piezoelectric actuator	Simultaneous measurement of forces/mechanics	Low throughput (single cell)
								Localized deformations	Natural cell-substrate interface not reachable
									2D
Thermoresponsive hydrogel^11^	600–800 nm	±100 nm	ms	13 pN–50 pN	0.5–2 pN/s	Extracellular	IR pulsed laser	Directly incorporated at the cell-substrate interface	Possible heating of sample due to IR laser
								High spatiotemporal control for activation	Low kinetic control
								Scalable (from subcellular to multiple cells and more)	

Although this study presents convincing demonstration of the potential of this tool, the development of the full potential of the method will motivate future research at different levels. More efficient motors to be activated at longer wavelengths^23^, understanding and quantification of rotation frequencies and force application rates, and their modulation by the design of the flexible space are relevant to open questions for improved performance. In addition, the study of force decay following illumination termination and its control through the relaxation mechanisms of the polymeric spacer offer additional ways to probe and understand the mechanical language in biological systems. Coupling to molecular force sensors (FRET tension probes^24–26^) could allow straightforward integration of force application and readout in simple additive steps. The flexibility of the design, not coupled to materials or special techniques, will allow extension of this technique to natural biointerfaces and in vivo contexts.

## Methods

### Preparation of motor/PEG/surface conjugate

N-hydroxysuccinimide (NHS) functionalized NEXTERION® slide H (Schott, 1070936) was used as substrate for cell experiments. NEXTERION® Slide H is coated with a non-fouling polymer layer and contains N-Hydroxysuccinimide (NHS) ester groups for reaction with amine-functionalized molecules.

The NEXTERION® slide H was incubated with 50 µL of 1 mM dibenzocyclooctyne-amine (DBCO-amine) solution in anhydrous DMSO for 1 h, and subsequently rinsed with Milli-Q water (200 µL) three times. The substrate was then incubated with 50 µL of a 0.5 mM solution in Milli-Q water of polymer-motor conjugate (or control polymer-motor conjugate). After 12 h, the solution was removed and the substrate was then immersed in O-(2-Azidoethyl)-O’-methyl-triethylene glycol (PEG-azide) (20 mM in water, 50 µL) for 0.5 h to block the unreacted DBCO groups, and subsequently washed with Milli-Q water (200 µL) three times. This substrate was immediately coupled to the biological ligand (next sections).

### Preparation of RGD/motor/PEG/surface conjugate

The motor/PEG/surface conjugate prepared as described in 1.1 was further incubated with cyclo(RGDfK-N_3_) solution (0.1 mg/mL in PBS, 50 µL, 0.1 mM) containing sodium ascorbate (1 mg/mL, 1 µL, 0.1 mM) and CuSO_4_·5H_2_O (1 mg/mL, 1 µL, 0.08 mM). The reaction was allowed to proceed for 6 h at room temperature. The modified substrate was rinsed with PBS (200 µL) three times. The slides were used for cell experiments immediately after modification.

### Preparation of α-CD3/motor/PEG/surface conjugate

The motor/PEG/surface conjugate prepared as described in 1.1 was further incubated with biotin-PEG_3_-N_3_ solution (1 mg/mL in water, 50 µL, 2.2 mM) containing sodium ascorbate (1 mg/mL, 1 µL, 0.1 mM) and CuSO_4_·5H_2_O (1 mg/mL, 1 µL, 0.08 mM). The reaction was allowed to proceed for 3 h at room temperature and rinsed with Milli-Q water three times. The substrate was then incubated with streptavidin (100 µg/mL in PBS, 50 µL) for 1 h at room temperature. After rinsed with Milli-Q water, the substrate was incubated with biotin anti-human CD3 Antibody (Biolegend Cat#317320) (50 µg/mL in PBS, 50 µL) for 12 h at 4 °C. The substrates were then rinsed with Ringer solution containing 0 mM Ca^2+^ (200 µL) three times. The slides were used for cell experiments immediately after preparation.

### Preparation of α-CD28/motor/PEG/surface conjugate

The motor/PEG/surface conjugate prepared as described in 1.1 was further incubated with biotin-PEG_3_-N_3_ solution (1 mg/mL in water, 50 µL, 2.2 mM) containing sodium ascorbate (1 mg/mL, 1 µL, 0.1 mM) and CuSO_4_·5H_2_O (1 mg/mL, 1 µL, 0.08 mM). The reaction was allowed to proceed for 3 h at room temperature and rinsed with Milli-Q water three times. The substrate was then incubated with streptavidin (100 µg/mL in PBS, 50 µL) for 1 h at room temperature. After rinsed with Milli-Q water, the substrate was incubated with biotin anti-human CD28 Antibody (Biolegend with Cat# 302904) (50 µg/mL in PBS, 50 µL) for 12 h at 4 °C. The substrates were then rinsed with Ringer solution containing 0 mM Ca^2+^ (200 µL) three times. The slide was used for cell experiments immediately after preparation.

### TPM experiment and preparation of DNA/motor/PEG/surface conjugate

The microfluidic channel was constructed by sandwiching a double-sided polyimide film (Kapton tape) between a previously modified motor/PEG NEXTERION® slide H and a microscopy slide. A 1 mm × 10 mm rectangular channel was cut into the Kapton tape using a CO_2_ laser cutter. Two 0.8-mm holes were drilled into the microscopy slide as in- and outlet for solutions. The tubing was connected to the in- and outlet via 10 µL Pipette tips gently pushed into the holes.

The DNA-tethers were attached to the motor molecules in a two-step process. First, a DNA-oligomer was covalently attached to the motor molecules providing anchoring points for the longer DNA construct that serves as a tether for the micro-beads. The 50 bp, 3′ azide terminated single strand DNA-oligomer 5′-CATCACCTTGCTGAACCTCAAATATCAAACCCTCAATCAATATCTGGTCA-3′) (Integrated DNA Technology) was covalently attached to the motor/PEG NEXTERION® slide H by incubating the channel with a 10 µM DNA-oligomer solution in nuclease-free water for 12 h in presence of CuSO_4_·5H_2_O and Na-ascorbate. Then, the channel was washed three times with 10 µL nuclease-free water and the channel walls were passivated with 10 mg/ml Western Blocking Reagent (Sigma-Aldrich) in PBS for 1 h. The blocking solution was replaced every 15 min. In the second step, a 2.7-kbp long DNA construct was attached to the surface wall via hybridization with the DNA-oligomers. The DNA construct was assembled from circular M13mp18 ssDNA (New England Biolabs) and functionalized with biotin to enable attachment of streptavidin-functionalized beads as described in ref. ^27^. The region complementary to the DNA-oligomer was left single-stranded to enable efficient hybridization. The DNA solution (7 µL, 100 pM) was pipetted into the channel and incubated for 12 h. Subsequently, streptavidin-coated beads (Dynabeads MyOne C1, ThermoFisher Scientific) were injected into the fluid channel for 5 min to attach to the DNA via specific binding of biotin and streptavidin. Previously, the beads were washed extensively and diluted to a concentration of 1 g/mL in PBS. After tethering, the chamber was flipped upside down and loose beads were washed out by applying a gentle fluid flow of 0.5 µL/min. The chamber was used immediately for experiments. The samples for the control experiments were prepared in the same way using the non-rotary motor (control).

### Cell culture and reagents

Fibroblast L929 cell line (ATCC CRL-636) was cultivated at 37 °C in 5% CO_2_ in RPMI medium (Gibco) supplemented with 10% fetal bovine serum (Invitrogen) and 1% P/S (Invitrogen). Cells were used between passages P4 and P16.

Mouse embryonic fibroblasts (MEFs) stably expressing paxillin-RFP^28^ were cultured in high-glucose DMEM (ThermoFisher) supplemented with 10% FBS (manufacturer), 2 mM GlutaMAX (Gibco), and 1% penicillin-streptomycin (manufacturer). For imaging experiments, the culture medium was replaced with phenol red-free DMEM Fluorobrite (Gibco), supplemented with the same supplements as above. For experiments with ROCK inhibitor Y27632 (10 µM), cells were pre-incubated for 30–60 min before imaging.

Jurkat T cells (DSMZ-German collection of microorganisms and cell cultures. ACC 282) were cultured in RPMI-1640 medium (ThermoFisher) with 10% FBS (ThermoFisher) and 1% penicillin-streptomycin (Gibco). Primary human CD4^+^ T cells were isolated by untouched CD4^+^ T-cell isolation kit (Miltenyi Biotec). Human CD4^+^ T cells were activated by Dynabeads Human T-Activator CD3/CD28 (ThermoFisher) and cultured in AIMV medium (ThermoFisher) with 10% FBS (ThermoFisher) and in presence of 33 U/ml human IL-2 (premium-grade, Miltenyi Biotec).

### Optimization of illumination conditions in MEFs

A UGA-42 Firefly point scanning laser device (RAPP Optoelectronic) with a 365-nm laser was coupled to an inverted epifluorescence Zeiss Axio Observer Z1 microscope. The laser focus was calibrated using a chromium calibration slide (RAPP Optoelectronic) prior to each experiment. At 50% duty cycle (the power used for the cell experiments and the minimal output we could measure using the laser meter), the power of the laser was 660 nW (measured using a laser meter from LABMASTER®) with a laser spot size of 4.4 μm^2^ with the usage of an OD3 filter. This results in an irradiance of 15 mW/cm^2^ (OD2: 150 mW/cm^2^). The substrate underneath the cell was illuminated using a custom circular ROI with a diameter of 8.86 μm (area = 62 μm^2^) by using the SysCon software. The ROI was scanned with 5 scans per illumination (Duration 234.75 ms; 5 runs/object 1234.75 ms), repeated every 20 s for duration of 15 min. The sample was irradiated simultaneously while acquiring RFP images for widefield fluorescence microscopy.

Initial exposure experiments were performed with a neutral density (ND) filter of OD2 (1% transmission) resulting in a light dose of 150 mW/cm^2^, with a cyclical illumination regime of one illumination pulse (40 scans per illumination) per 20 s (Supplementary Fig. 3). All cells monitored showed immediate cell retraction (within 6 min) of the illuminated cell areas, on both motor substrate (8 out of 8) and control substrate (4 out of 4). Cells did not show this behavior when lowering the laser transmission by using a stronger ND filter (OD3—0.1% transmission), although cell retraction was still observed on motor substrates (86%, 6 out of 7 cells) and control substrates (60%, 3 out of 5). Only when additionally lowering the amount of scans per illumination pulse (5 scans per illumination) did most cells on both motor (75%, 9 out of 12) and control substrates (80%, 8 out of 10) not show any cell retraction. These illumination conditions were used for the experiments.

### Live-cell imaging for focal adhesions

Cells expressing intermediate to low levels of paxillin were chosen to avoid overexpression artifacts. Cells were imaged on an inverted epifluorescence Zeiss Axio Observer Z1 microscope using a Plan-Apo ×40 Oil Objective (NA = 1.4) and 1.6 Tubelens, coupled to an Axiocam 506 CCD camera. Samples were illuminated using the Colibri 530 nm LED coupled with the DsRed filterset (set 43) with 8% power of 530 nm LED for 500 ms exposure time for each time point. In addition, brightfield images were taken (100 ms exposure) with the TL lamp set to 3.5 V. Images were obtained with 2 × 2 binning and 2x analog gain every 30 s for 15 min. Images were captured using the Zen Blue software.

### Focal adhesion image processing and analysis

All image processing was done using the Fiji distribution of ImageJ^29^ with the Morpholib plugin library^30^. For the reference images (Fig. 2a and b), paxillin-RFP images were first filtered with a Gaussian Blur (sigma = 1), then the Grays LUT was inverted and contrast was enhanced.

To analyze illuminated versus non-illuminated areas, first ROIs containing several FAs for these areas were cropped, and then each ROI was separately processed and analyzed. For focal adhesion segmentation, a custom ImageJ macro was developed. In short, images were first filtered using a Gaussian blur (sigma = 1.5), then a 3D White top Hat filter (element = cube, xyz-radii were all set at 5) from the Morpholib plugin was applied, after which background was subtracted using a rolling ball of 20. Then a mask was generated using the Otsu auto-threshold. Small noise particles were then excluded by Area opening (pixel size = 20). The total size of all FAs was then measured using the resulting mask. The relative increase in total FA area was determined as total FA size at *t*/total FA size at *t*_0_.

### Optimization of irradiation conditions in T cells

For T-cell activation, we employed a Zeiss Cell Observer HS system with a ×20 alpha objective and an AxioCam MRm Rev. 3. The DAPI channel (100% power, 10 mW/cm^2^) was used for UV irradiation. A sequence of ten UV pulses with a duration of 1, 0.5, or 2 s were applied within 1 min to activate rotation of the motor. The Ca^2+^ fluorescent signal was followed for 15–20 min.

### Calcium imaging and data analysis

Jurkat T cells and primary T cells were loaded with Fluo-4/AM (1 µM) in serum-free RPMI-1640 media at room temperature for 30 min. Afterward, cells were pelleted by centrifugation, followed by one wash using Ringer’s solution containing 0 mM Ca^2+^. Then cells were settled on the motor-linked substrates for 8 min before measurement. Afterward, the cells were washed once with Ringer’s solution containing 0 mM Ca^2+^. Then the same volume of Ringer’s solution containing 2 mM Ca^2+^ was added prior to the start of calcium imaging. Fifteen minutes later, UV illumination was conducted for 1 min, followed by another 15 min of imaging. To detect a general capability of the cells to induce Ca^2+^ influx, 1 µM thapsigargin is added 10 min after UV illumination.

For calcium imaging, we employed a Zeiss Cell Observer HS system with a ×20 alpha objective and an AxioCam MRm Rev. 3. During the experiment, the fluorescence of Fluo-4 was acquired with a 38HE filterset every 5 s. Mean fluorescence intensity of Fluo-4 was quantified with ImageJ. The cell spreading areas were determined using the Wand (tracing) Tool in ImageJ and then quantified with ImageJ. The peak of Ca^2+^ influx (ΔPeak) is the maximum relative the fluorescence of Fluo-4 (normalized to baseline before UV illumination) from first UV pulse to 120 s.

### Particle tracking in the flow cell

All Flow Cell measurements were performed with a syringe pump (AL-1000 World Precision Instruments, Sarasota, USA) equipped with a 3-ml syringe (BD Diagnostics). A CCD camera (ImagingSource, Bremen, Deutschland) with 3.072 × 2.048 pixels mounted on a standard optical microscope with a ×50 magnification was used for recording the movement of surface-tethered beads in one field of view. Prior to every experiment, the zero position of the beads was determined by recording the Brownian motion of the beads for 60 s with a sampling rate of 5 fps. Subsequently, a constant flow of 2–15 µL/min was applied for 8 min. After 2 min of constant flow, the UV LED with a maximum intensity at a wavelength of 365 nm and a power of ~0.75 W/cm^2^ was switched on for 4 min. Force calibration was performed in DNA overstretching experiments with linear flow ramps from 0 to 1000 µL/min controlled via the syringe pump. The actual flow velocity was monitored via the height of the fluid in the reservoir (Fig. 3a).

### TPM data analysis

The digital videos were analyzed using the open-source software *ImageJ 1.50i* (Wayne Rasband, National Institute of Health, USA) and a *Particle Tracker* plugin for *ImageJ* written by Sbalzarini and Koumoutsakos. The *Particle Tracker* provides $x$ and $y$ positions of each individual bead for every frame. The *ImageJ* software includes all trajectories into a table that was further analyzed in MATLAB regarding their Brownian motion and bead displacement under fluid flow. Before data analysis, all trajectories were drift-corrected using a master curve generated by averaging the trajectories of three to five immobile beads^31^. The heterogeneity of the molecular motor attachment results in a statistical distribution of biotin-binding sites leading to variety of tethered particle motion. The Brownian motion of all beads in one frame was recorded for 60 s prior to every measurement. Only trajectories with a RMS position fluctuation above 100 nm were included in further analysis to omit unspecific beads with non-specific attachment. This lower limit reflects an effective tether length *L* = (3*〈RMS〉²)/2*b*^32^ of above 375 nm assuming a Kuhn-length of $b=$40 nm.

### Surface preparation for AFM

The Au(111) was purchased from Arrandee Metal GmbH + Co. KG in Germany and used as substrate for AFM experiments. Before functionalization with a self-assembled monolayer, the sample was annealed five times for 1 min in a butane flame to achieve flat gold terraces. Then, the surface was immersed in 1-Azidoundecan-11-thiol (1 mM in ethanol) overnight and used on the same day in AFM experiments.

### Cantilever functionalization

The triangular gold-coated Si_3_N_4_ AFM cantilever (NPG-10 D, Bruker, France) used for AFM experiments were functionalized in three steps. In the first step, a SAM was immobilized using the thiol functionality when incubating the surface for 24 h in 1 mM HS-C11-EG6-OCH2-COOH dissolved in ethanol. In a second step, the carboxylgroup of the SAM was activated using EDC/NHS for 15 min followed by incubation in 1 mM DBCO-amine in DMSO for 2 h. In the third step, the motor was attached to the SAM using the azide-functionality of the PEG chains at room temperature. The cantilever was prepared freshly and used within 3 days in AFM experiments.

### AFM experiments

AFM measurements were performed with a Nanowizard 3 setup (JPK Instruments, Berlin, Germany) with the motor-functionalized cantilever on a SAM coated gold surface at room temperature. The AFM cantilever with nominal normal spring constant of 0.06 N/m has been using the thermal noise analysis. In AFM force spectroscopy experiments, the AFM tip is approached to the surface and held in contact for 60 s with a setpoint of 500 pN to allow a covalent reaction between the PEG end group attached to the motor molecule and the N_3_ functionality of the SAM covering to the gold surface. The reaction was carried out in the presence of copper sulfate and sodium ascorbate. After immobilizing the motor molecule between AFM tip and surface, the AFM tip is retracted for 40 nm with a velocity of 0.02 µm/s to stretch the PEG5000 chains attached to the motor molecules, followed by a pause of 180 s at a constant height of 40 nm above the surface. After a waiting time of 30 s, the UV light was switched on for a duration of 90 s and the normal force was monitored. After the pause time of 180 s, the AFM tip was fully retracted from the surface.

### Reporting summary

Further information on research design is available in the Nature Research Reporting Summary linked to this article.

## Supplementary information

Supplementary Information

Reporting Summary

## Data Availability

The authors declare that all the datasets generated during and/or analyzed during the current study are available from the corresponding author on reasonable request.
