# Supplementary material for: Optoregulated force application to cellular receptors using molecular motors
Source: Nat Commun. 2021 Jun 11;12:3580. doi: 10.1038/s41467-021-23815-4 (PMC8196032; doi:10.1038/s41467-021-23815-4)
Supplement: Supplementary file 1 — Supplementary Information [file 41467_2021_23815_MOESM1_ESM.pdf]

Supplementary Information

Optoregulated force application to cellular receptors using molecular motors

Yijun Zheng, Mitchell K.L. Han, Renping Zhao, Johanna Blass, Jingnan Zhang, Dennis W. Zhou, Jean-Rémy Colard-Itté, Damien Dattler, Arzu Çolak, Markus Hoth, Andrés J. García, Bin Qu, Roland Bennewitz, Nicolas Giuseppone, Aránzazu del Campo

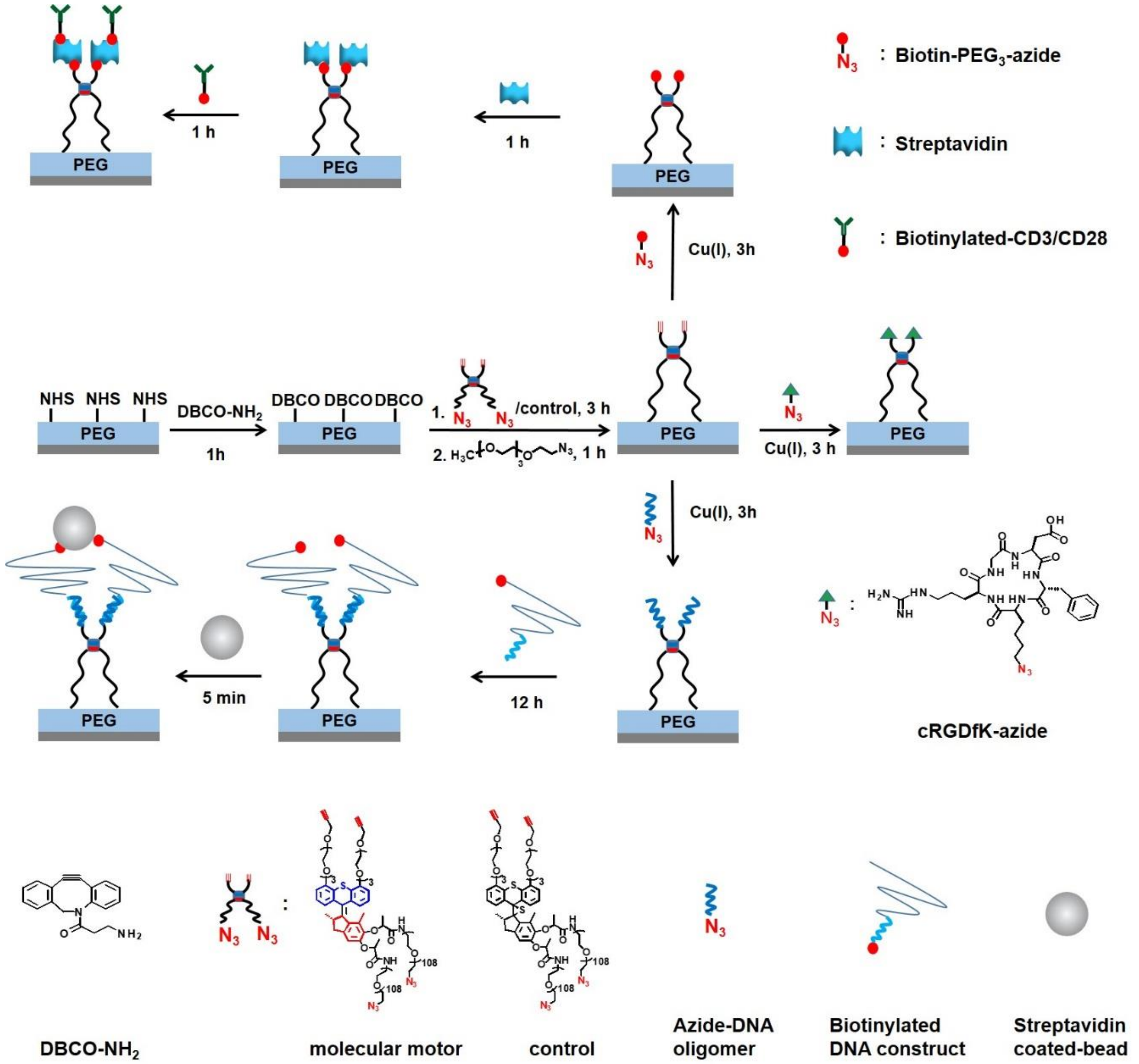

Supplementary Figure 1. Schematic illustration of the preparation of ligand (orDNA)/motor/PEG/surface conjugates through a multiple-step procedure. See methods for more details.

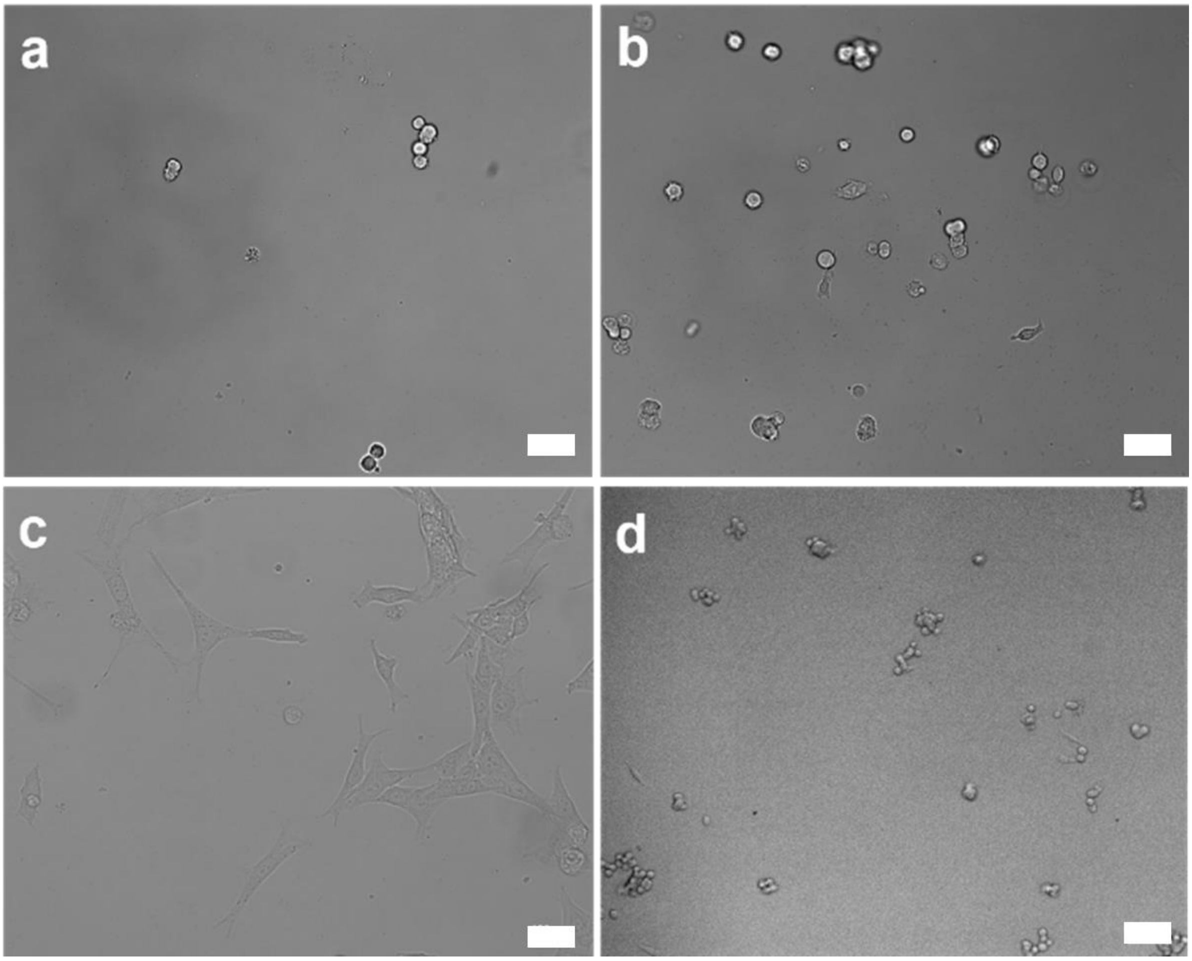

Supplementary Figure 2. Cell spreading on motor substrates. L929 fibroblasts incubated on (a) motor/PEG/surface and (b) on substrates incubated with RGD without the motor for 24 hours (scalebars in (a) and (b): 50 µm). (c) Fibroblasts incubated on RGD/motor/PEG/surface after 7 days (scalebar: 100 µm). (d) Fibroblasts incubated on RDG/motor/PEG/surface for 24 hours (scalebar: 50 µm).

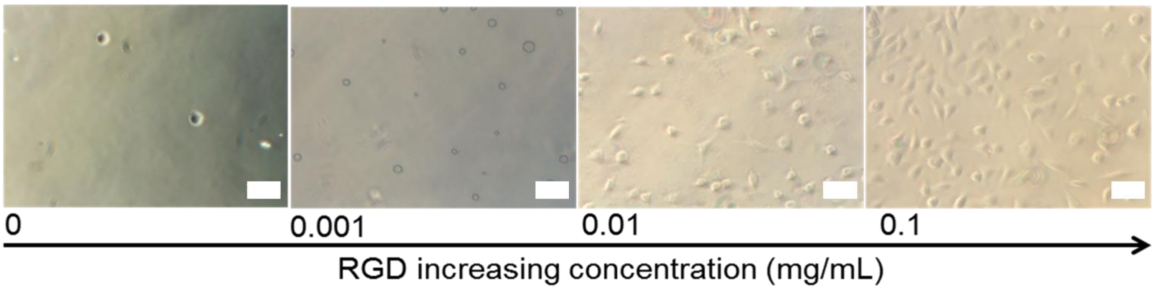

Supplementary Figure 3: Representative bright field images of L929 cells cultured on RGD/motor/PEG/surfaces at increasing concentrations of RGD peptide with a seeding number of 4000 cells/cm^2 after incubation for 24 hours (scalebar: 100 µm).

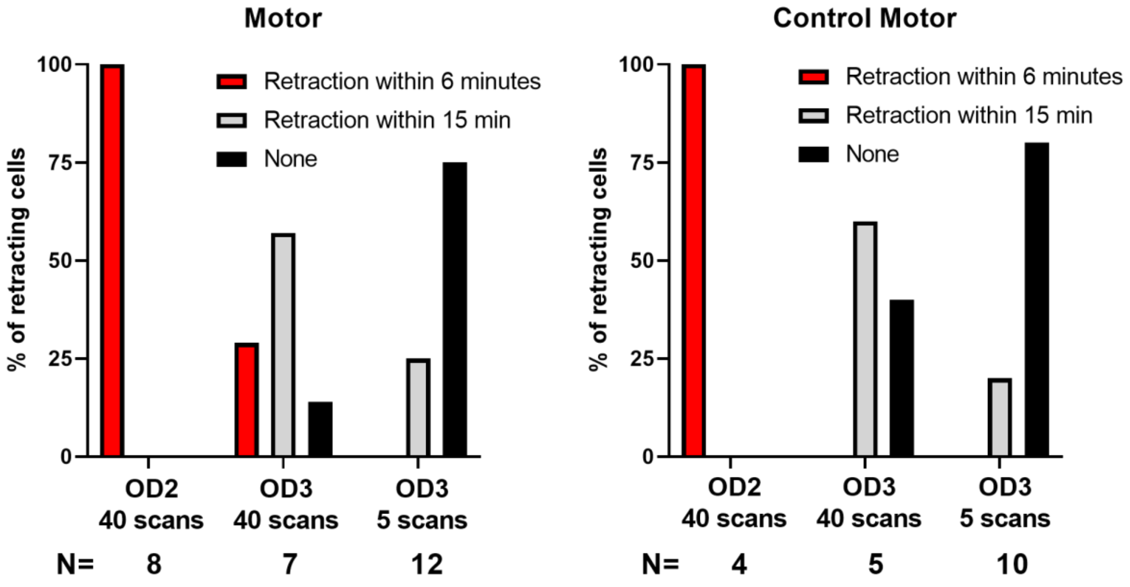

Supplementary Figure 4. Increasing UV-illumination dosage leads to cell retraction. Motor substrates (left) or control motor substrates (right) were illuminated with a scanning UV-laser (365 nm – 5 runs per 20s). Cell areas within the field of illumination were analyzed for cell retraction (removal of cell arm from illuminated area). OD is a neutral density filter blocking light transmission form the laser source, with OD2 and OD3 resulting in 1% and 0.1% light transmission respectively. *N* represents the number of cells analyzed for each condition.

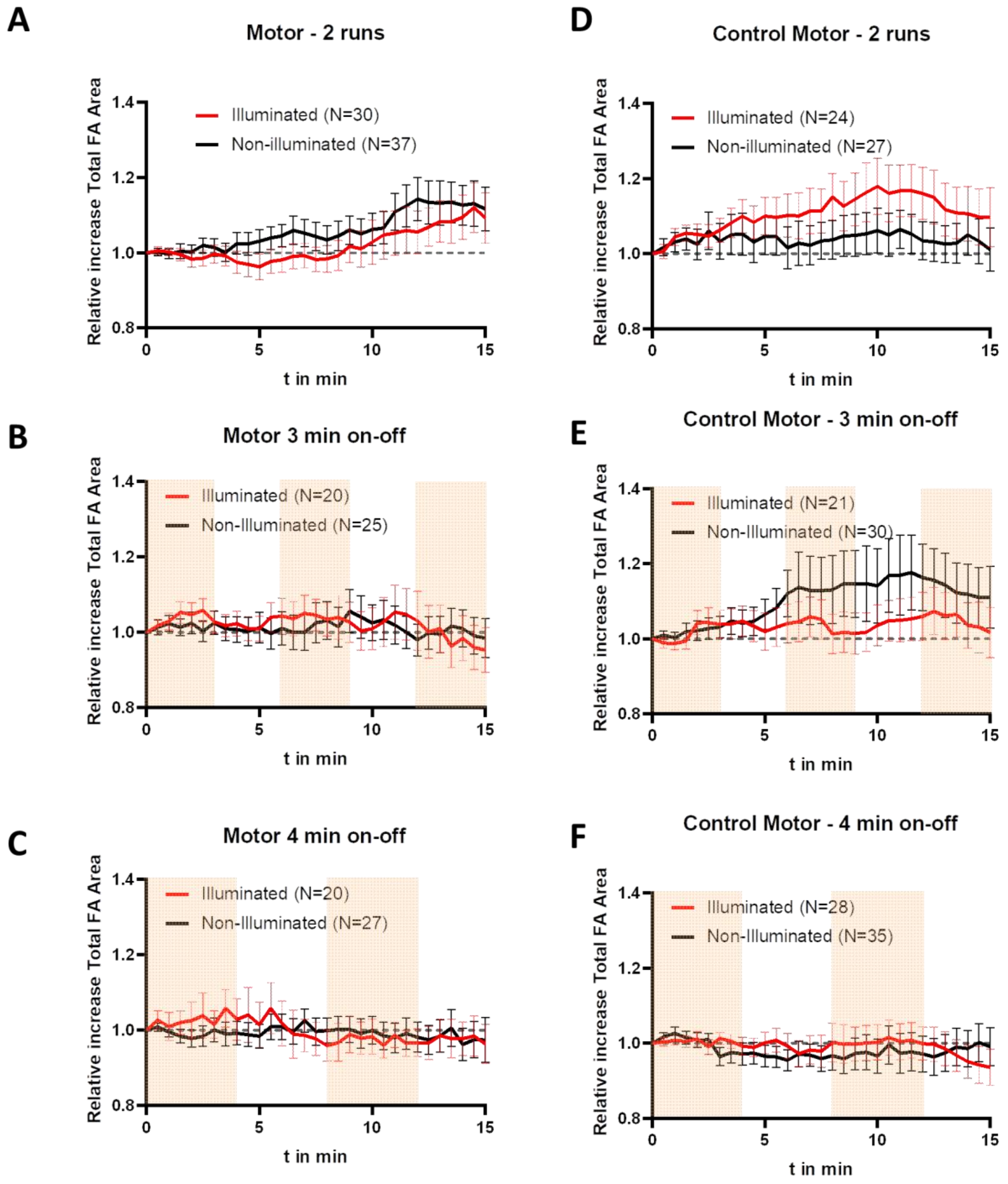

Supplementary Figure 5. Differing illumination doses which did not lead to Focal Adhesion growth. Plots represent the mean ± s.e.m. of relative increase in Total Focal Adhesion Area analyzed within n# of ROIs of cells seeded on motor (a-c) or control motor (d-f) substrates irradiated with different irradiation programs. (a,d) 2 scans per 20s irradiation. (b,e) 5 scans per 20s -3 min on – 3 min off. Illuminated times are marked in orange. (c,f) 5 scans per 20s - 4 min on – 4 min off. Illuminated times are marked in orange. All data was generated from three independent experiments, except (c) which was generated from two independent experiments.

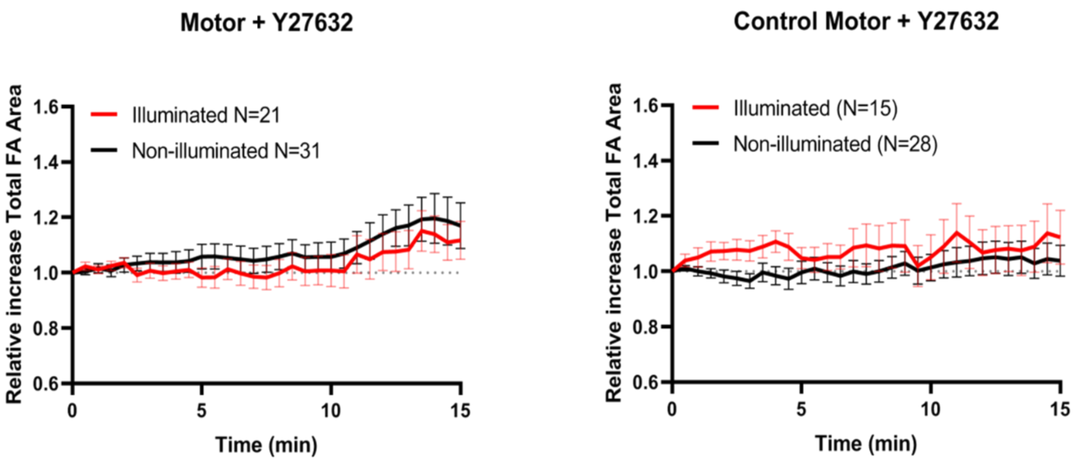

Supplementary Figure 6. ROCK inhibition perturbs motor-induced FA activation. Cells were pre-incubated with ROCK inhibitor Y27632 (10 µM) for 30-50 min. Plots represent the mean ± s.e.m. of relative increase in Total Focal Adhesion Area of Focal adhesions analyzed within *n#* of ROIs of 21 cells from three independent experiments seeded on motor (left) or of 15 cells from two independent experiments on control motor (right) substrates.

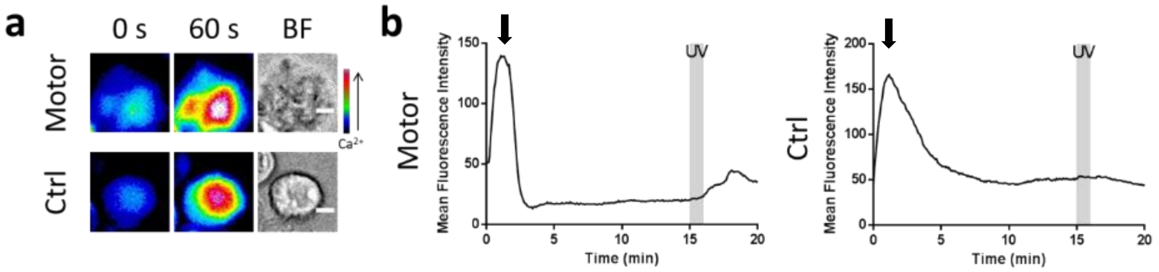

Supplementary Figure 7. Activation of Fluo-4-AM loaded Jurkat T cells on the αCD3/motor/PEG/surface. Control experiments using the non-rotary motor were also run in parallel. The cells were incubated in 0 mM Ca^{2+} Ringer's solution at room temperature for 8 min. Then the same volume of 2 mM Ca^{2+} Ringer's solution were added (0 min). At the time point of 15 min, the cells were illuminated by UV. A sequence of ten UV pulses with a duration of 1 second were applied within 1 minute and Fluo-4 fluorescent signal was followed for 20 mins. (a) Heat map of Fluo-4 fluorescence intensity. BF stands for bright field. Scale bars are 5 μm. (b) Analysis of intracellular Ca^{2+} dynamics in Jurkat T cells shown in (a).

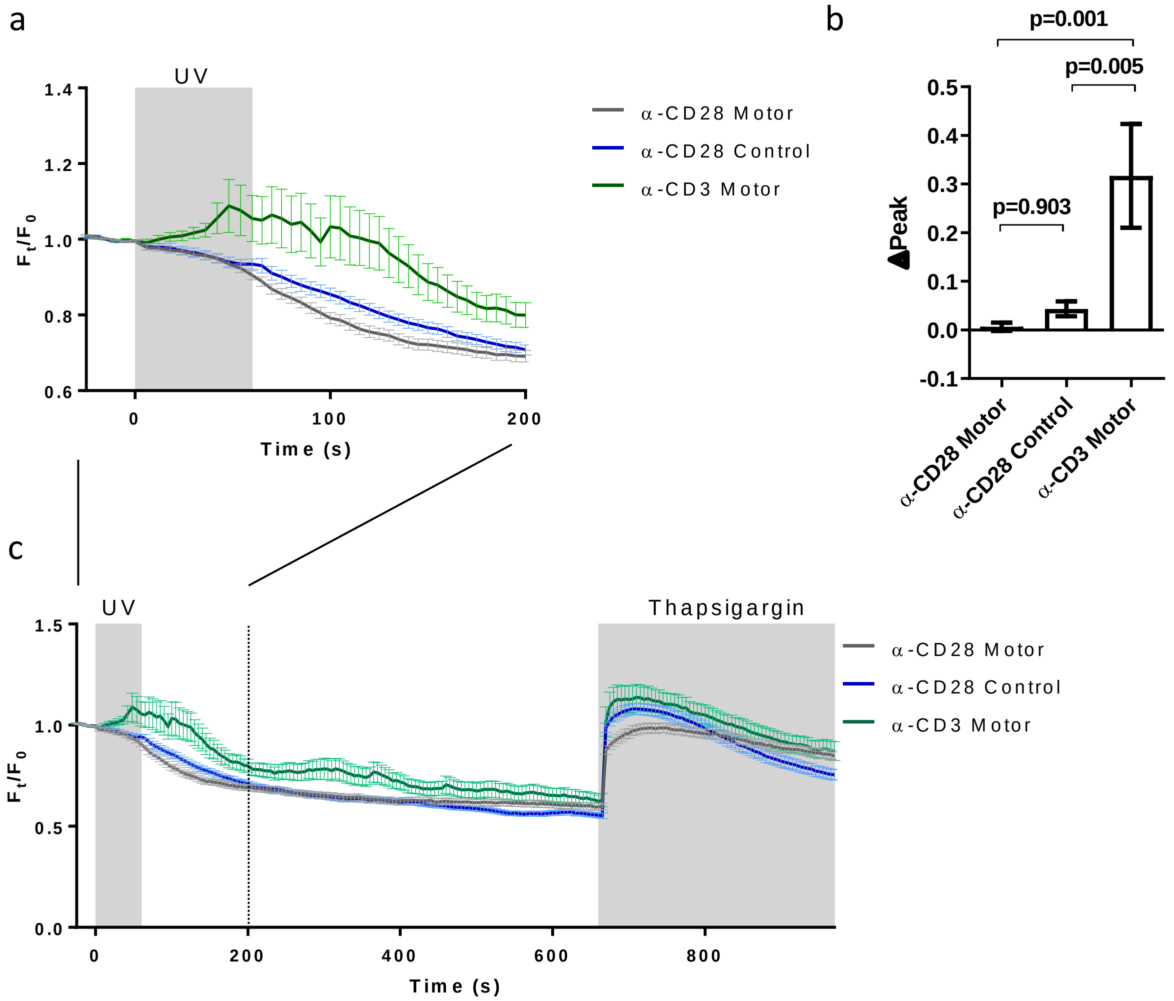

Supplementary Figure 8. Response of T cells on αCD28/motor/PEG/surfaces and controls. Jurkat T cells loaded with Fluo-4-AM were seeded on the substrate for 15 min before UV illumination. 10 pulses (1 s duration) of UV light were applied for a total duration of 1 min. (a) αCD28/motor does not induce Ca^{2+} influx. αCD3/motor serves as a positive control. (b) Quantification of the maximum Ca^{2+} influx (ΔPeak) in a. (c) UV illumination does not hamper the capacity of Ca^{2+} influx. 10 min After UV illumination, the cells were activated by thapsigargin (1 μM) to induce the maximum Ca^{2+} influx. The results were from 4 independent experiments (α-CD28 Motor, *n*=115 cells; α-CD28 Control, *n*=120 cells; α-CD3 Motor, *n*=120 cells) and represented as mean ± s.e.m.

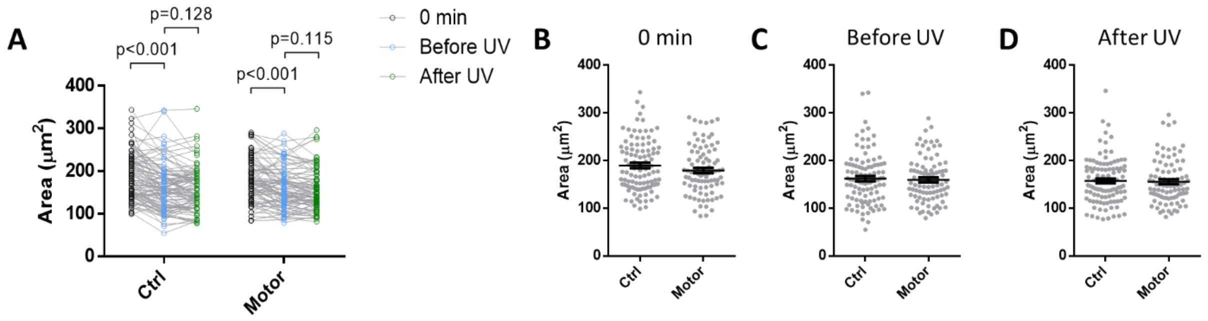

Supplementary Figure 9. Mechanical force applied by the motor does not change the contact area at the IS. Fluo-4 loaded Jurkat cells were seeded on αCD3 modified substrates. The CD3 antibody was linked with the surface via non-rotary motor (control, 100 cells) or rotary motor (88 cells). The contact areas were determined at three time points: the beginning of measurement (0 min – black dots), before (blue dots) and after UV illumination (green dots). (a) The contact area at the IS does not change upon rotation of the motor. Paired t-test was used for the statistics. (b-d) No difference in the contact area is found between control and motors. The results were from 6 independent experiments and represented as mean ± s.e.m.

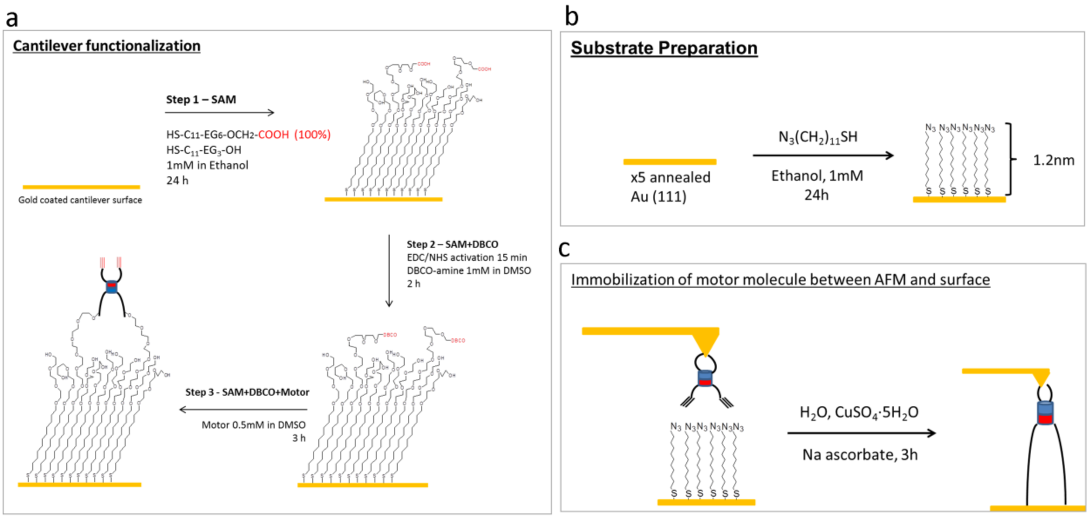

Supplementary Figure 10: Steps of AFM cantilever (a) and gold surface (b) functionalization, see methods section for details. (c) Covalently coupling the motor molecule to the N_3-covered gold surface, see methods section for details.

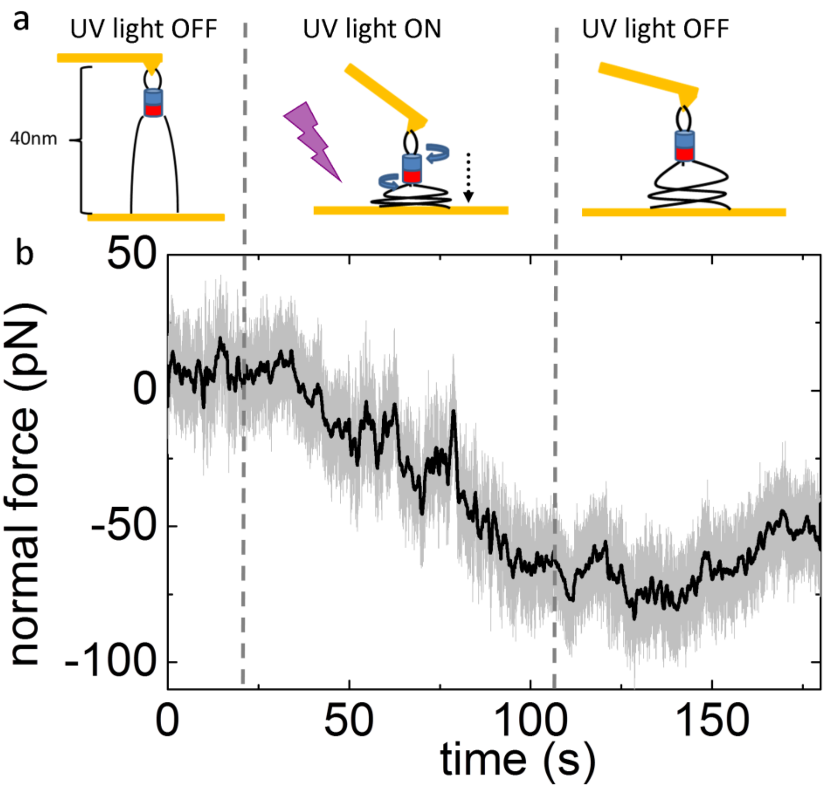

Supplementary Figure 11: (a) Sketch of an AFM force-spectroscopy experiment using a motor-functionalized AFM tip. (b) Exemplary AFM force-spectroscopy experiment where the AFM tip is held 40 nm above the surface. Upon UV stimulation from 30s to 120s, a monotonous increase of negative force up to about 80 pN is observed, followed by a slight decrease of negative force when the UV light is switched OFF after 120s.

Supplementary Discussion

Force measurements by observation of tethered particle motion

Creep in tethered particle motion

After onset and stabilization of flow (1 min), the tethered beads are typically displaced by a few hundred nanometers. The variation in initial displacement can be explained by a variation of the exact number of tethers and by the distribution of tether attachment on the Nexterion substrate. We then observed creep of the beads position, i.e. beads were continuously displaced in the flow direction. We attribute the creep to relaxation processes in the substrate gel and to a detachment and reattachment of the non-covalent biotin-streptavidin bonds of DNA tethers to the bead surfaces.

Method and scaling arguments

In the cell experiments, motor-chain conjugates were coupled to a Nexterion PEG-hydrogel, resulting in a random distribution of effective tether length between receptors and surface. The heterogeneous distribution of tether lengths is expected to generate a distribution of pulling forces applied to cellular focal adhesions. We introduce the video microscopy of tethered particle motion (TPM)[4] as a novel approach that allows investigation of hundreds of different motor-chain conjugates in parallel. Under flow and light exposure, this method allows parallel observation of drag force-induced displacement and light-induced retraction of hundreds of beads by optical microscopy. Other single molecule experiments such as atomic force microscopy (AFM) or optical or magnetic tweezers require the statistical evaluation of many repetitions to achieve piconewton force resolution. The time necessary to link single molecules and to record repeated force measurements impedes the investigation of heterogeneous systems, where many locations have to be probed to determine the distribution of mechanical properties.

In our TPM experiment, molecular motors are attached to the surface of the channel using the same PEG hydrogel functionalization as in the cell experiments. Beads with a diameter of 500 nm, comparable to the size of a focal adhesion, are tethered to the molecular motors by DNA chains with a length of 1.7 µm. The beads are connected to a small number of tethers, which resemble the attachment of focal adhesions by multiple motor-chain conjugates in the cell experiment. We use a DNA construct[5] which offers great flexibility in the attachment of functional end groups for linking the chains.

The observation of motion of tethered microparticles by video microscopy requires longer spacer chains than the ones used in the cell experiments. The contour length of the DNA construct (1700 nm) is about 53 times that of the PEG_{5000} linkers (32 nm) in the cell experiment. The persistence length of the DNA construct (12.6 nm) is about 36 times that of PEG_{5000} chains (0.35 nm[6]). Thus, the overall coiling geometry the two experiments is scaled

and we can expect a similar relative reduction in extension upon twisting of polymer pairs in the entropic low-force regime. In absolute numbers, the same rotational twist induced by the motor molecule will induce a 50 times smaller length reduction in the 50 times shorter PEG chains, but this relative extension will produce a 36 times larger force than in the DNA experiments as all entropic forces for the same relative polymer extension scale as $k_{\mathrm{B}}T/P$, where P is the persistence length[7]. This scaling ultimately needs confirmation for the case of twisted pairs of polymer chains by an adequate model or simulations.

The DNA chains are constructed by hybridizing an ssDNA with a set of matching oligomers with a length of 60 bp each. The persistence length of this construct was determined by fitting an extensible worm-like chain model to the extension-force curves of single dsDNA constructs. The extension-force curves were recorded by analyzing the displacements of tethered beads in an increasing flow. The value of 12.6 nm for the persistence length is significantly lower than the values around 50 nm typically reported for dsDNA[8]. We attribute the difference to the high number of nicks every 60 base pairs, i.e. of breaks in the phosphate backbone of one strand of the dsDNA.

AFM Force Spectroscopy Experiments on motor molecules with PEG_{5000} linkers

We performed AFM force spectroscopy experiments on the molecular motors with PEG_{5000} linkers immobilized between the cantilever and a flat gold surface. The AFM cantilever was functionalized with the molecular motors and a flat gold surface was coated with N_3-SAM, see Supplementary Figure 10a,b. The functionalization is thus the same as in the cell experiments.

AFM force spectroscopy experiments were performed in three steps. First, the motor molecule attached to the AFM tip and was covalently coupled to the surface using a click chemistry reaction of the free end of the PEG_{5000} chains and the N_3-covered surface, see Figure 9c. Second, the AFM tip was retracted for 40 nm, the estimated distance of stretched PEG_{5000} linkers. In the third step, the actual force spectroscopy experiment, the AFM tip was held in a constant distance of 40 nm above the surface, see Figure 11a. After a waiting time of 30s, the UV light was switched ON for 90s and the variation in normal force was monitored. One exemplary force-spectroscopy experiment where the changes in force are measured over time is shown in Supplementary Figure 11b. Upon UV stimulation from 30s to 120s, a monotonous increase of negative force up to about 80 pN is observed. The decrease of force is caused by a bending of the cantilever towards the surface, indicating a photo-initiated response of the motor when coiling the PEG chains immobilized between the AFM tip and the surface. The force value is in the range which is expected to activate mechanotrsanduction in cells. It also confirms the scaling argument (a factor of about 36) for the relation between forces measured upon twisting entangled PEG5000 linkers and forces measured in tethered-particle motion experiments upon twisting of entangled DNA constructs. After ending the UV stimulation, a slight decrease of negative force is observed, presumably due to the relaxation of coiled PEG chains.

Supplementary References

1 Matthews, B. D., Overby, D. R., Mannix, R. & Ingber, D. E. Cellular adaptation to mechanical stress: role of integrins, Rho, cytoskeletal tension and mechanosensitive ion channels. *Journal of Cell Science* **119**, 508, doi:10.1242/jcs.02760 (2006).

2 Kadem, L. F. *et al.* High-Frequency Mechanostimulation of Cell Adhesion. *Angewandte Chemie International Edition* **56**, 225-229, doi:10.1002/anie.201609483 (2017).

3 Liu, Z. *et al.* Nanoscale optomechanical actuators for controlling mechanotransduction in living cells. *Nat Methods* **13**, 143-146, doi:10.1038/nmeth.3689 (2016).

4 Nelson, P. C. *et al.* Tethered Particle Motion as a Diagnostic of DNA Tether Length. *The Journal of Physical Chemistry B* **110**, 17260-17267, doi:10.1021/jp0630673 (2006).

5 Koussa, M. A., Halvorsen, K., Ward, A. & Wong, W. P. DNA nanoswitches: a quantitative platform for gel-based biomolecular interaction analysis. *Nat Methods* **12**, 123-126, doi:10.1038/nmeth.3209 (2015).

6 Oesterhelt, F., Rief, M. & Gaub, H. E. Single molecule force spectroscopy by AFM indicates helical structure of poly(ethylene-glycol) in water. *New Journal of Physics* **1**, 6-6, doi:10.1088/1367-2630/1/1/006 (1999).

7 Petrosyan, R. Improved approximations for some polymer extension models. *Rheologica Acta* **56**, 21-26, doi:10.1007/s00397-016-0977-9 (2017).

8 Bustamante, C., Smith, S. B., Liphardt, J. & Smith, D. Single-molecule studies of DNA mechanics. *Curr Opin Struct Biol* **10**, 279-285, doi:10.1016/s0959-440x(00)00085-3 (2000).
