# Supplementary material for: Optoregulated force application to cellular receptors using molecular motors
Source: Nat Commun. 2021 Jun 11;12:3580. doi: 10.1038/s41467-021-23815-4 (PMC8196032; doi:10.1038/s41467-021-23815-4)
Supplement: Supplementary file 2 — Reporting Summary [file 41467_2021_23815_MOESM2_ESM.pdf]

nature research

Corresponding author(s): Aránzazu del Campo

Last updated by author(s): Mar 12, 2021

Reporting Summary

Nature Research wishes to improve the reproducibility of the work that we publish. This form provides structure for consistency and transparency in reporting. For further information on Nature Research policies, see Authors & Referees and the Editorial Policy Checklist .

Statistics

For all statistical analyses, confirm that the following items are present in the figure legend, table legend, main text, or Methods section.

n/a	Confirmed	
☐	☒	The exact sample size (n) for each experimental group/condition, given as a discrete number and unit of measurement
☐	☒	A statement on whether measurements were taken from distinct samples or whether the same sample was measured repeatedly
☐	☒	The statistical test(s) used AND whether they are one- or two-sided<br>*Only common tests should be described solely by name; describe more complex techniques in the Methods section.*
☒	☐	A description of all covariates tested
☒	☐	A description of any assumptions or corrections, such as tests of normality and adjustment for multiple comparisons
☐	☒	A full description of the statistical parameters including central tendency (e.g. means) or other basic estimates (e.g. regression coefficient) AND variation (e.g. standard deviation) or associated estimates of uncertainty (e.g. confidence intervals)
☐	☒	For null hypothesis testing, the test statistic (e.g. F, t, r) with confidence intervals, effect sizes, degrees of freedom and P value noted<br>*Give P values as exact values whenever suitable.*
☒	☐	For Bayesian analysis, information on the choice of priors and Markov chain Monte Carlo settings
☒	☐	For hierarchical and complex designs, identification of the appropriate level for tests and full reporting of outcomes
☒	☐	Estimates of effect sizes (e.g. Cohen's d, Pearson's r), indicating how they were calculated

Our web collection on statistics for biologists contains articles on many of the points above.

Software and code

Policy information about availability of computer code

Data collection	Zeiss Zen Blue software. AxioVision 4.1.8.
Data analysis	FIJI with the Morpholib and Particle Tracker plugins. Graphpad Prism 8. MATLAB. Graphpad Prism 6. ImageJ with Particle Tracker plugins.

For manuscripts utilizing custom algorithms or software that are central to the research but not yet described in published literature, software must be made available to editors/reviewers. We strongly encourage code deposition in a community repository (e.g. GitHub). See the Nature Research guidelines for submitting code & software for further information.

Data

Policy information about availability of data

All manuscripts must include a data availability statement. This statement should provide the following information, where applicable:

- Accession codes, unique identifiers, or web links for publicly available datasets
- A list of figures that have associated raw data
- A description of any restrictions on data availability

All raw data is available upon request.

Field-specific reporting

Please select the one below that is the best fit for your research. If you are not sure, read the appropriate sections before making your selection.

☒ Life sciences ☐ Behavioural & social sciences ☐ Ecological, evolutionary & environmental sciences

For a reference copy of the document with all sections, see nature.com/documents/nr-reporting-summary-flat.pdf

Life sciences study design

All studies must disclose on these points even when the disclosure is negative.

Sample size	Sample sizes were not pre-determined but based on previous literature in the same subject area and from experience relating to the observations with the assays performed.
Data exclusions	For the FA size analysis, some data values were excluded due to image processing artifacts
Replication	Reproducibility was verified by performing each experiment independently several times.
Randomization	Randomization was not required (repeated measures of the same cells before and after intervention).<br>For T cell activation experiment, the analyzed cells were randomly selected.
Blinding	Blinding was not possible due to active device setup for the intervention.

Reporting for specific materials, systems and methods

We require information from authors about some types of materials, experimental systems and methods used in many studies. Here, indicate whether each material, system or method listed is relevant to your study. If you are not sure if a list item applies to your research, read the appropriate section before selecting a response.

Materials & experimental systems

n/a	Involved in the study	
☐	☒	Antibodies
☐	☒	Eukaryotic cell lines
☒	☐	Palaeontology
☒	☐	Animals and other organisms
☒	☐	Human research participants
☒	☐	Clinical data

Methods

n/a	Involved in the study	
☒	☐	ChIP-seq
☒	☐	Flow cytometry
☒	☐	MRI-based neuroimaging

Antibodies

Antibodies used	Biotin-CD3 antibody (Biolegend Cat#317320), Biotin-CD28 antibody (Biolegend with Cat#302904)
Validation	Both antibodies are Flow Cytometry Quality tested. Manufacturer's website lists many references, but Zhang DKY, et al. 2020. Nature Protocols. 15(3):773-798 is valid for both.

Eukaryotic cell lines

Policy information about cell lines

Cell line source(s)	L929 (ATCC CRL-636)<br>MEF paxillin-RFP (Zhou et al. 2017 - https://doi.org/10.1091/mbc.e17-02-0116)<br>Jurkat T-cells (DSMZ-German collection of microorganisms and cell cultures. ACC 282)
Authentication	MEFs expressing paxillin-RFP showed the expected structures and cell morphology.
Mycoplasma contamination	Cell lines were tested negative for mycoplasma contamination
Commonly misidentified lines (See ICLAC register)	The cell lines used are not commonly misidentified
